# Optimization of culture conditions for the derivation and propagation of baboon (Papio anubis) induced pluripotent stem cells

**DOI:** 10.1371/journal.pone.0193195

**Published:** 2018-03-01

**Authors:** Christopher S. Navara, Shital Chaudhari, John R. McCarrey

**Affiliations:** 1 Department of Biology, University of Texas at San Antonio, San Antonio, Texas, United States of America; 2 San Antonio Cellular Therapeutics Institute, University of Texas at San Antonio, San Antonio, Texas, United States of America; University of Texas at Austin Dell Medical School, UNITED STATES

## Abstract

Induced pluripotent stem cells (iPSCs) offer the possibility of cell replacement therapies using patient-matched cells to treat otherwise intractable diseases and debilitations. To successfully realize this potential, several factors must be optimized including i) selection of the appropriate cell type and numbers to transplant, ii) determination of the means of transplantation and the location into which the transplanted cells should be delivered, and iii) demonstration of the safety and efficacy of the cell replacement protocol to mitigate each targeted disease state. A majority of diseases or debilitations likely to be targeted by cell-based therapeutic approaches represent complex conditions or physiologies manifest predominantly in primates including humans. Nonhuman primates afford the most clinically relevant model system for biomedical studies and testing of cell-based therapies. Baboons have 92% genomic similarity with humans overall and especially significant similarities in their immunogenetic system, rendering this species a particularly valuable model for testing procedures involving cell transplants into living individuals. To maximize the utility of the baboon model, standardized protocols must be developed for the derivation of induced pluripotent stem cells from living adults and the long-term maintenance of these cells in culture. Here we tested four commercially available culture systems (ReproFF, mTeSR1, E8 and Pluristem) for competence to maintain baboon iPSCs in a pluripotent state over multiple passages, and to support the derivation of new lines of baboon iPSCs. Of these four media only Pluristem was able to maintain baboon pluripotency as assessed by morphological characteristics, immunocytochemistry and RT-qPCR. Pluristem also facilitated the derivation of new lines of iPSCs from adult baboon somatic cells, which had previously not been accomplished. We derived multiple iPS cell lines from adult baboon peripheral blood mononuclear cells cultured in Pluristem. These were validated by expression of the pluripotency markers OCT4, NANOG, SOX2, SSEA4 and TRA181, as well as the ability to differentiate into tissues from all three germ layers when injected into immunocompromised mice. These findings further advance the utility of the baboon as an ideal preclinical model system for optimizing iPS cell-based, patient-specific replacement therapies in humans.

## Introduction

The isolation and culture of human embryonic stem cells (hESCs) in 1998 [1] ushered in a promising new age in cell-based therapeutics. The ability of these pluripotent cells to form all tissues of the body meant that novel treatments could be envisioned for a number of otherwise intractable diseases including neurodegenerative diseases, diabetes, heart disease, rheumatoid arthritis, macular degeneration, infertility and spinal cord injury, among others. However multiple key challenges have hindered the optimization of these cell-based therapies and their translation to the clinic, including the fact that the use of embryonic stem cells (ESCs) typically requires the destruction of embryos, and that transplants involving derivatives of ESCs require an allograft that can potentially induce immunorejection or that may require a lifelong immunosuppression regime [2]. The derivation of induced pluripotent cells (iPSCs) in 2006 [3–5] appeared to solve both problems simultaneously, because iPSCs can be derived from somatic cells recovered from each patient yielding a “patient-specific” approach that i) avoids the need to destroy embryos, and ii) facilitates therapeutic use of an autograft that should minimize immune response, although this is still in question and may depend on both the type of cell transplanted and the location of the transplant [6–10].

Beyond these concerns, the safe translation of stem cell-based therapies to the clinic raises several additional challenges including i) determination of the optimal type of cells to transplant (e.g. fully differentiated cells or progenitor cells), ii) determination of the optimal route of delivery of cells designed to treat each specific condition, iii) optimization of post-transplant survival and propagation of cells, iv) validation of proper ongoing gene expression and epigenetic programming in the transplanted cells, v) confirmation that the transplanted cells display proper function including regulated actions, vi) determination of the extent to which each iPSC-based therapy can mitigate the specific disorder to be treated, both acutely and chronically, and vii) validation of the absence of any associated undesirable off-target effects such as tumorigenesis, immune rejection or aberrant differentiation. These objectives can only be addressed *in vivo*, however direct transition to clinical trials runs the risk of adverse outcomes in patients. Therefore, a clinically relevant animal model is needed to facilitate preclinical studies in a manner that will maximally inform subsequent efforts to translate the use of safe and effective cell-based therapeutic approaches to the clinic. Old world monkeys are closely related to humans in genomic sequence, physiology, anatomy (including neuroanatomy), size, and immunogenetic organization, and, in contrast to the great apes, their availability, size and cost facilitate preclinical studies that can be used to optimize the efficacy and safety of cell-based therapies leading to the effective translation of these methodologies to the clinic.

In particular, the olive baboon, *Papio anubis*, shares important similarities with humans, including overall anatomical, physiological, developmental and genomic features [11]; VandeBerg et al., 2009). The baboon provides an optimal preclinical model for studies of cell-based therapeutic approaches to the treatment of neurodegenerative diseases [12] based on its large gyrencephalic brain [13], the human-like ratio of white to grey matter [14], and cerebral microvasculature similar to that found in the human brain [15]. Additionally, the baboon closely resembles the human in developmental processes, behavioral characteristics, lifespan (up to 45 years) and genomic sequence (the baboon genome sequence is ~ 92% similar to the human genome sequence) [16, 17].

With respect to modeling a particular neurodegenerative disease such as Parkinson’s Disease (PD), the baboon affords additional features that mimic the human and are particularly advantageous for preclinical studies, including the physical separation of the nuclei of the striatum which is the target of dopaminergic neuron projections, motor and non-motor manifestations of an MPTP-induced model of Parkinsonian symptoms, extrastriatal innervation from the SNc, and the prevalence of type D2 dopamine receptors in the frontal cortex [12].

Finally, and of critical importance to the development of cell-based transplantation therapies for any disease or disability, the baboon immune system more faithfully resembles the human immune system than does that found in either rhesus monkeys or mice [18–21]. Thus, given the recently imposed prohibitions regarding the use of chimpanzees in biomedical research, (NIH, 2016) the baboon now provides the single most relevant model in which to test the extent of any immunogenicity of transplanted cells [22] as well as to test the efficacy and safety of cell-based therapies to mitigate PD or other maladies (Grow et al., 2016b).

While extensive progress has been made in the ability to manipulate human iPSCs in culture, including the generation of “footprint-free” iPSCs [23, 24], and/or directed differentiation to yield specific, functional somatic cell types including dopaminergic neurons [25], cardiomyocytes [26, 27], and β-islet cells [28–30] among others, the efficacy and safety of the use of these cells for cell-based therapeutic applications in living individuals remains to be optimized. To test the ultimate clinical approach of auto-transplantation of patient-specific differentiated derivatives of human iPSCs back into the patient from whom the iPSCs were derived, an optimal preclinical model must recapitulate derivation of iPSCs from a living individual, followed by maintenance and differentiation of these cells in culture, and subsequent auto-transplantation of the differentiated cells back into the same living animal. To this end, we previously isolated baboon iPSCs (biPSCs) [31] and demonstrated that these can be induced to differentiate into clinically relevant cell types in culture, including dopaminergic neurons similar to those that become defective in PD patients [32]. However, to optimize the baboon model for preclinical studies of cell-based therapeutic approaches, it is important to maximize the efficiency and consistency of methods for the derivation and maintenance of biPSCs in a pluripotent state. Thus, the ability to derive footprint-free iPSCs from accessible somatic cells of living baboons, maintain these cells in a pluripotent state, induce these cells to differentiate into specific cell types, and then transplant the differentiated derivatives back into the same, live animal will provide the most relevant preclinical animal model.

In our experience, baboon pluripotent cells, including both bESCs [33] and biPSCs [31], have proven to be particularly challenging to derive and maintain in culture relative to pluripotent cells from other mammalian species including the mouse [34], rhesus monkey [35, 36], and human [37–39]. Specifically, baboon pluripotent cells have shown a propensity to undergo spontaneous differentiation in culture. Thus, although we have previously succeeded in deriving biPSCs from baboon fibroblasts (Navara et al., 2013), the efficiency of this success was approximately 1000-fold less than that we have experienced with the derivation of iPSCs from human fibroblasts. Finally, our previously reported success in deriving biPSCs was limited to those derived from fetal fibroblasts, which is not relevant to the clinical objective of modeling iPSC derivation from adult patients.

To optimize the utility of the baboon model for studies of the efficacy and safety of cell-based therapies to be translated for use in patients, we sought to identify standardized culture conditions that will support the routine derivation and maintenance of biPSCs in a more consistent manner than that we have achieved to date. A previous study by the International Stem Cell Initiative demonstrated that commercially produced media often perform more consistently than the same formulation derived in an individual laboratory, perhaps because of the large batch preparation and the more stringent quality control standards associated with commercial production of media [40]. Therefore, we conducted a screen of several commercially available pluripotent stem cell media for efficacy in promoting the long-term maintenance of biPSCs in an undifferentiated state in culture. Our second objective was to optimize a protocol for the derivation of biPSCs from an adult somatic cell type–preferably a cell type that can be relatively easily recovered from live adult animals. We found that the culture conditions we optimized for the maintenance of baboon pluripotent cells can also be used to support the derivation of biPSCs from peripheral blood mononuclear cells (PBMCs). These advances now facilitate the use of the baboon as an optimal preclinical model for testing the efficacy and safety of cell-based therapeutic approaches to the study, diagnosis and/or treatment of disease or debilitations.

## Materials and methods

### Cell culture

We previously described a baseline culture method for baboon iPSCs [31]. Briefly, mitotically inactivated mouse embryonic feeders (MEFs, EMD Millipore, Billerica, MA) were seeded onto gelatin coated tissue culture plates at a density of 2.6 x 10^4^ cells/cm^2^ in MEF medium (DMEM, 10% Fetal Bovine Serum, 1 mM GlutaMax, 0.1 mM nonessential amino acids, 100 U/mL penicillin, 100 μg/mL streptomycin [all from ThermoFisher Scientific, Waltham, MA]). Two days after seeding, the medium was replaced with iPSC media (80% KO-DMEM, 20% knockout serum replacer, 1 mM GlutaMax, 0.1 mM nonessential amino acids, 100 U/mL penicillin, 100 μg/mL streptomycin, and 4 ng/mL FGF2 (ThermoFisher Scientific). biPSCs were then seeded onto the MEFs and resulting colonies were manually passaged once every 5–7 days using a fire-polished glass Pasteur pipette. Media was replaced every 24 hours and cells were maintained at 37°C/5% CO_2_. Use of this baseline media formula was compared to four other commercially available media–ReproFF (Stemgent, Lexington, MA), mTeSR1 and TeSR-E8 (both from Stem Cell Technologies, Cambridge MA) and PluriStem (EMD Millipore), following the corresponding manufacturer’s directions.

### Comparison of commercially available media

Baboon iPSCs cultured in conditioned media were passaged onto MEF’s in conditioned media as described above. 48 hours after passaging cells were switched to one of the commercial media or maintained in conditioned media. When the cells reached the point of next passage they were analyzed for morphological characteristics indicative of pluripotent versus differentiated cells, and processed for immunocytochemistry or RT-PCR as described below.

### Derivation of footprint free iPSCs

To derive footprint free biPSCs from PBMCs, we used the Cytotune 2.0 kit (ThermoFisher Scientific) following the manufacturer’s instructions. Briefly, 5 ml peripheral blood was collected from adult baboons at the Texas Biomedical Research Institute (TBRI, San Antonio, TX) at either the time of necropsy of animals sacrificed for other purposes, or from live baboons following TBRI-IACUC approved protocols. PBMCs were collected from baboon blood using BD vacutainer CPT cell isolation tubes (Becton Dickinson, Franklin Lakes, NJ). Tubes containing blood were centrifuged at 1800 x g for 30 minutes. The PBMC cell layer was collected and washed twice in Stempro 34 medium (ThermoFisher Scientific) and the cells were plated into 24 well plates at a density of 2–5 x 10^5^ cells/well. PBMCs were fed with StemPro-34 medium (ThermoFisher Scientific) containing stem cell factor (SCF, 100ng/ml), FLT-3 (100ng/ml), IL-3 (20ng/ml) and IL-6(20ng/ml). Isolated PBMCs were cultured for four days with daily media changes. On the fourth day sendai viruses carrying sequences encoding pluripotency factors (KOS, hMYC and hKLF4) at an MOI of 5:5:3 respectively were added to the culture media. To maximize transduction, the cells and virus were centrifuged at 1000 x g for 30 min. After 24 hr of culture the virus was washed out of the media and the cells were placed into low attachment 24 well plates for 48 hr before being plated onto inactivated MEFs in StemPro-34 media containing cytokines. 24 hours after plating, the media was changed to StemPro-34 without cytokines. On day 7 the cells were switched to Pluristem media and cultured until colonies appeared–typically 13–17 days later. At approximately 28 days after plating, colonies were passaged onto new feeders and the resulting biPSC lines were maintained as described above.

### Confirmation of the loss of episomal sendai virus and lack of genomic integration

We isolated RNA and produced cDNA from baboon iPSCs as described below. We used validated Taqman probes designed against the reprogramming vectors (SEV, Mr04269880_mr, KOS, Mr04421257_mr, KLF4, Mr04421256_mr, and cMYK, Mr04269876_mr, all from Invitrogen). Vector probes were compared against a housekeeping gene (HPRT1, Hs99999909_m1) using the ΔΔCt method.

We isolated genomic DNA from the baboon iPSCs using a QIAamp DNA micro kit (Qiagen, Germantown, MD) according to manufacturers directions. Genomic DNA was then subjected to qPCRas above with the exception that the comparison gene utilized a Taqman probe known to cross react with genomic DNA (NANOG, Hs02387400_g1, Invitrogen)

### Immunocytochemistry (ICC)

Cells were grown on plastic coverslips (Electron Microscopy Sciences, Hatfield, PA). At the indicated times, media was aspirated and the coverslips were washed with phosphate buffered saline composed of 136.94 mM NaCl, 9.74 mM sodium phosphate dibasic, 1.70 mM potassium phosphate monobasic, and 2.65 mM KCl in 18 MΩ H_2_O (PBS). The coverslips were then placed into 2% paraformaldehyde for 30 minutes at 37°C and washed three times in PBS with 0.5% triton-X-100 (PBS-TX-100). Cell membranes were permeabilized by incubation in PBS-TX-100 for 15 minutes at room temperature. Primary antibodies were applied overnight at 4°C. A list of antibodies and concentrations used is provided in S1 Table. After washing three times with PBS-TX-100, cover slips were incubated with an appropriate fluorescently labeled secondary antibody (1:100) for one hour at room temperature. Coverslips were then washed and mounted onto microscope slides, coated with ProLong Gold Antifade with DAPI (ThermoFisher Scientific), covered with a cover glass and allowed to cure for 24 hours at room temperature before imaging.

Imaging was performed using a Deltavision Personal DV system (GE Healthcare Life Sciences, Pittsburgh, PA) equipped with high numerical aperture objectives and a digital CoolSnap HQ2 cooled CCD Camera. Images were prepared for publication using SoftWorx (Applied Precision, Issaquah, WA), ImageJ [41], and Adobe Photoshop (Adobe Systems, San Jose CA).

### RNA isolation and RT-PCR

Cells were lysed and RNA was isolated using the TRIZOL® Reagent (ThermoFisher Scientific) followed by chloroform-mediated phase separation. RNA was precipitated using 100% isopropanol and washed with 75% ethanol. All RNA isolates were treated with an RNA clean-up kit (Ambion, [ThermoFisher Scientific) with DNase to remove any genomic DNA contamination. RNA was validated for purity and concentration using a NanoDrop spectrophotometer (ThermoFisher Scientific). 1 μg of the RNA isolate was converted to cDNA using a First Strand cDNA Synthesis kit according to the manufacturer’s directions (ThermoFisher Scientific). For each sample, one additional cDNA synthesis reaction was prepared without reverse transcriptase enzyme to serve as a No-RT negative control.

### RT-qPCR

100 ng of cDNA was loaded into each well of a 96-well plate containing 250 nM forward and reverse primers (see S2 Table for primer sequences), 10 μL of 2x Power SYBR master mix, and RNase-free H_2_O to a final volume of 20μL. The reaction was carried out in an Applied Biosystems (ThermoFisher Scientific) 7900 HT real-time thermal cycler and the results were processed using the Applied Biosystems RQ manager software. Each assay was performed in triplicate, and the results were averaged, and then normalized to an endogenous control (B2M) as described [32]. Comparisons were made across experimental conditions using the ΔΔC_T_ method [42, 43] and statistical comparisons were made using REST 2009 software [44]. P-values less than .05 were considered statistically significant. Data is displayed as the log_2_ of the fold change in normalized expression.

### Karyotype analysis

To ensure that each newly derived biPSC cell line possessed the appropriate chromosome number, all cell lines were outsourced for karyotype analysis. Cells lines were manually passaged into MEF-containing T-25 flasks and when confluent the flasks were sent to Cell Line Genetics (Madison, WI) for karyotyping.

### Teratoma formation and histological confirmation of pluripotency

All rodent research was reviewed and approved by the Institutional Animal Care and Use Committee of the University of Texas at San Antonio (assurance D16-00357). Every effort was made to alleviate suffering. To confirm pluripotency, one million biPSCs (derived as above, in 100 μl) were mixed 2:1 (vol/vol) with Matrigel and injected subcutaneously into immunocompromised mice (male, NOD.Cg-Rag1tmMomIL2rgtm1Wjl/SzJ, 7 weeks of age, Jackson Labs). For these brief injections UTSA veterinary staff determined no anesthesia was required. Animals were examined daily for adverse reactions and no adverse reactions aside from tumor formation were observed. Humane endpoints were followed as in UTSA policy #IACP 012. Animals were housed singly following injection in SPF conditions in a ventilated rack. When tumors reached 10mm in diameter or after 12 weeks, mice were sacrificed by CO_2_ asphixiation (20% displacement/minute) and the tumors excised. Tumors were fixed in 4% Formaldehyde in PBS and shipped to the Histology and Micro-Imaging Core (HMC) at Magee-Womens Research Institute where they were processed for histology and examined by a qualified pathologist to confirm the presence of cell types from all three germ layers. A total of five mice were used for these experiments.

### In vitro differentiation into the three germ lineages

We used the STEMdiff trilineage differentiation kit (Stemcell Technologies) following manufaturer’s directions to differentiate baboon iPSCs into ectoderm, endoderm and mesoderm. Briefly, cells were isolated with Accutase (Stemcell Technologies), counted with a hemacytometer and plated at 200,000 cells/cm2 (ectoderm and endoderm) or 50,000 cells/cm2 (mesoderm). Appropriate media was changed daily and cells were harvested on Day 5 (endoderm, and mesoderm) or Day 7 (ectoderm). RNA was collected as above and converted to cDNA. Primers for OCT4, SOX2, NES, PAX6, SOX17, FOXA2 and T were used to direct the appropriate tissues. Primer sequences can be found in S2 Table.

## Results

In our experience baboon pluripotent stem cells have been more difficult to derive and maintain than those from the mouse, human or rhesus monkey. For this reason we sought to improve and optimize methods for the derivation and maintenance of biPSCs. We previously maintained these cells under rich culture conditions, including growth on mouse embryonic feeders (MEFs) in media preconditioned by MEFs (Conditioned Media as described [31]). To optimize the use of the baboon as a preclinical model system, our objective was to develop efficient, standardized culture conditions for the derivation and maintenance of iPSCs from this species.

We first compared the growth of our existing lines of biPSCs maintained in conditioned (baseline) media to that of the same lines of biPSCs maintained in four different commercially available media–i) ReproFF, a serum-free proprietary media supplemented with bFGF, ii) mTeSR1, a semi-defined serum free culture medium containing 16 ingredients including bovine serum albumin (S3 Table, iii) TeSR-Essential 8 (E8), a completely defined culture medium with just eight components (S3 Table), and iv) Pluristem, a proprietary culture medium. Baboon iPSCs were expanded in each medium following the corresponding manufacturer’s suggested protocols, except that all were used in conjunction with inactivated MEFs as we have not successfully used feeder-free culture for baboon pluripotent stem cells. All media were changed daily to minimize effects of variable media stability.

**Fig 1:**
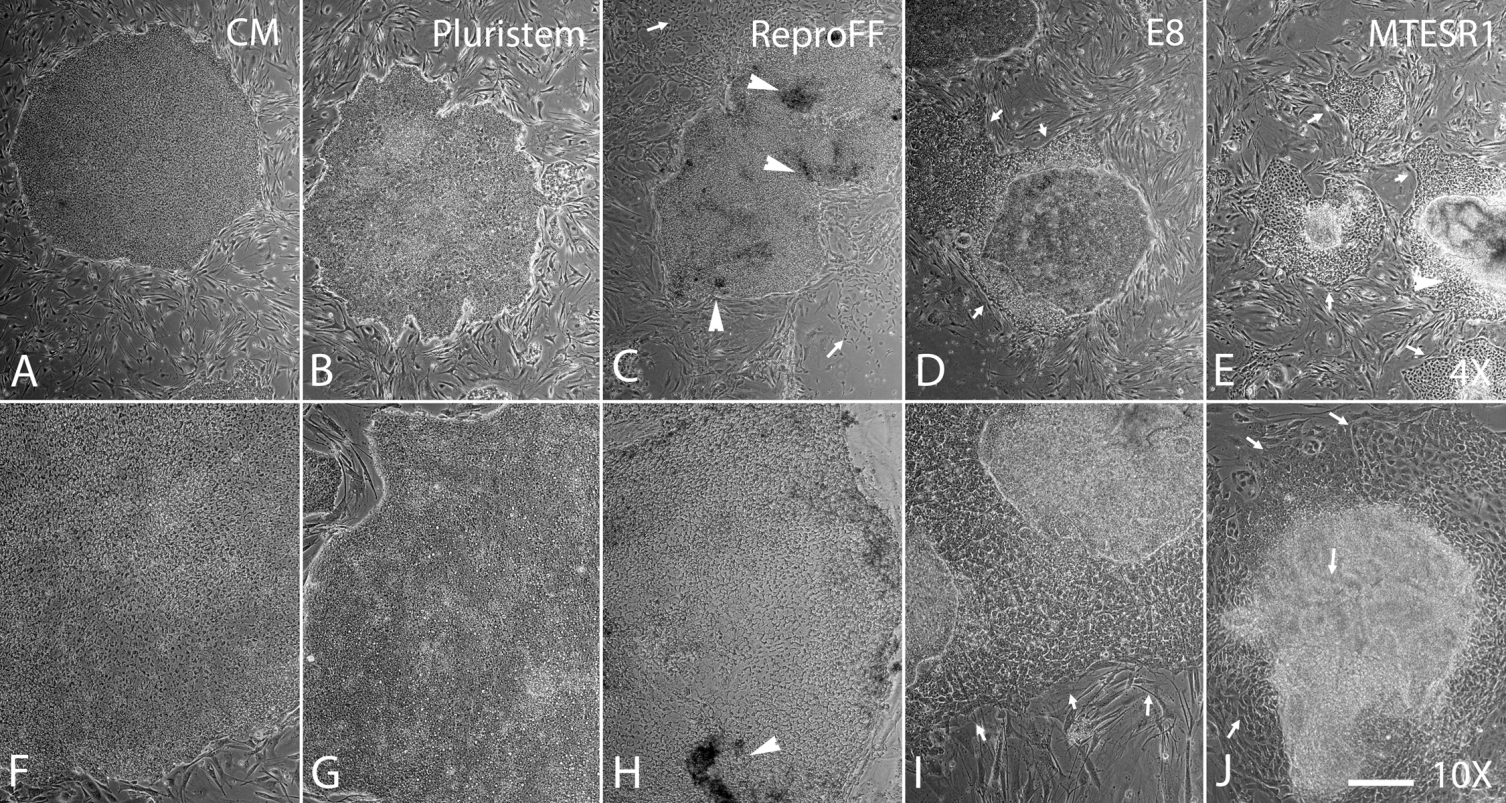
Comparison of culture conditions. Baboon iPSCs cultured on MEFs in conditioned media maintained closely packed cells with well-defined colony borders (A, F). Similar results were observed with iPSCs cultured in Pluristem (B, G). iPSCs cultured in ReproFF (C, H), mTeSR-1 (D, I) or mTeSR-E8 (E, J) showed signs of differentiation including dark areas (white arrowheads) and expanding differentiating zones (white arrows).

Baboon iPSCs grown in conditioned media display characteristic well defined colony borders and tightly packed cells (Fig 1A and 1F). Cells grown in Pluristem media shared these characteristics (Fig 1B and 1G) and could be maintained in this condition over multiple passages (greater than 30 to date). By contrast biPSCs grown in ReproFF (Fig 1C and 1H), TeSR-E8 (Fig 1D and 1I) or mTeSR1 (Fig 1E and 1J) showed signs of differentiation within four days of switching to those respective media, including dark areas within colonies (Fig 1C and 1H arrowheads) and expansive differentiated areas adjacent to colonies (arrows in Fig 1D, 1E, 1I and 1J). Cells cultured in these media could not be maintained in an undifferentiated state for longer than two passages.

**Fig 2:**
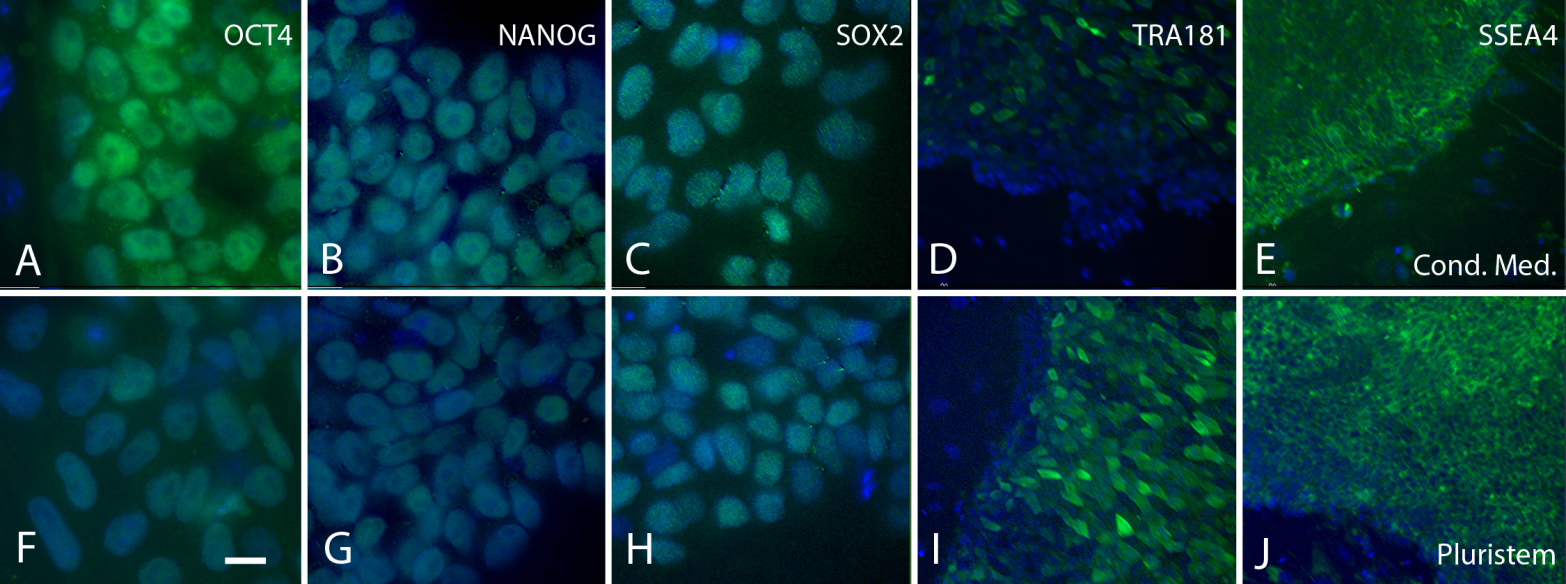
Immunocytochemical localization of pluripotency associated factors. As evaluated by immunocytochemical staining, baboon iPSCs cultured in conditioned media and Pluristem had similar expression levels and subcellular distribution of OCT4 (A,F, Green), NANOG (B,G, Green), SOX2 (C,H, Green), TRA181 (D,I, Green) and SSEA4 (E,J, Green) Blue = DNA, Bar = 20 μm.

**Fig 3:**
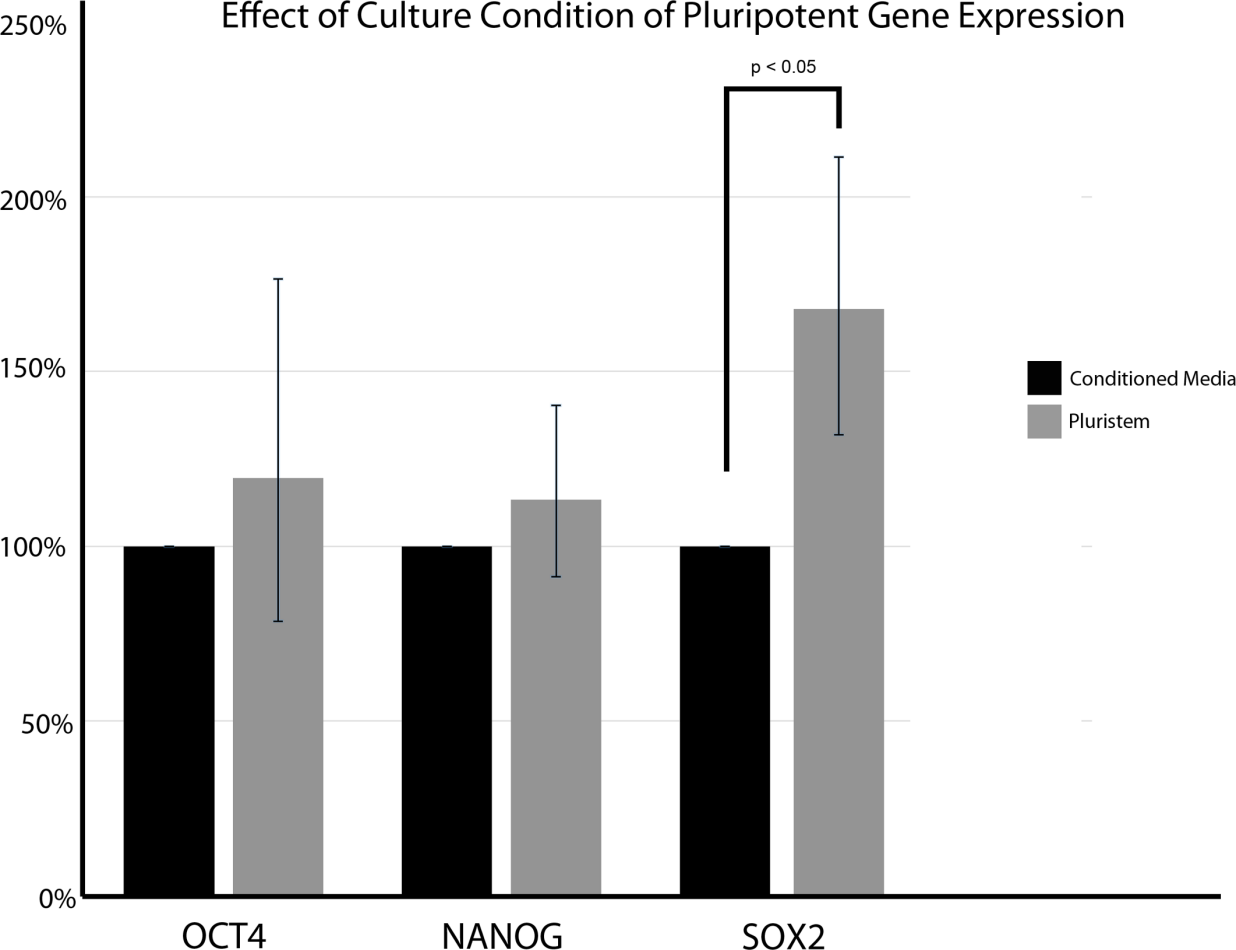
RT-qPCR of pluripotency factors. The expression of OCT4 and NANOG are similar between baboon iPSCs cultured in conditioned media and in Pluristem. The expression of SOX2 was significantly higher in iPSCs cultured in Pluristem. Bars = SEM p< 0.05.

We further characterized biPSCs maintained in Pluristem media compared to those maintained in our baseline conditioned media by immunocytochemistry which showed that cells maintained in each media displayed comparable levels and subcellular localization of the pluripotent transcription factors OCT4 (Fig 2A and 2F), NANOG (Fig 2B and 2G), and SOX2 (Fig 2C and 2H), and of the cell surface markers TRA181 (Fig 2D and 2J) and SSEA4 (Fig 2E and 2K). We next quantified expression of transcripts encoding the core pluripotency transcription factors (OCT4, SOX2 and NANOG) using qRT-PCR (Fig 3). Culture of cells in Pluristem led to expression of *OCT4* and *NANOG* transcripts at levels equal to those observed in biPSCs cultured in conditioned baseline media, however levels of *SOX2* transcripts were higher in cells cultured in Pluristem relative to those in cells cultured in our conditioned baseline media. These results indicate that Pluristem can sustain biPSCs in a pluripotent state over multiple passages at least as well as our baseline conditioned media.

Having established these standardized conditions for the maintenance of biPSCs in culture, we next sought to test the use of similar culture conditions for the derivation of new lines of biPSCs. For clinical use, a protocol facilitating derivation of iPSCs from somatic cells that are easily accessible from individuals of any age will be optimal. We tested the derivation of biPSCs from two somatic cell types easily accessible from adult baboons–skin fibroblasts and PBMCs. Although adult baboon fibroblasts remained refractory to reprogramming in our hands, even when cultured in Pluristem media, we found that PBMCs can be effectively reprogrammed in this medium. Thus we pursued optimization of a protocol for reprogramming PBMCs because this somatic cell type is easily accessible from adult baboons via a minimally invasive blood draw, and so provides an ideal model system for testing therapeutic applications of iPS cells.

**Fig 4:**
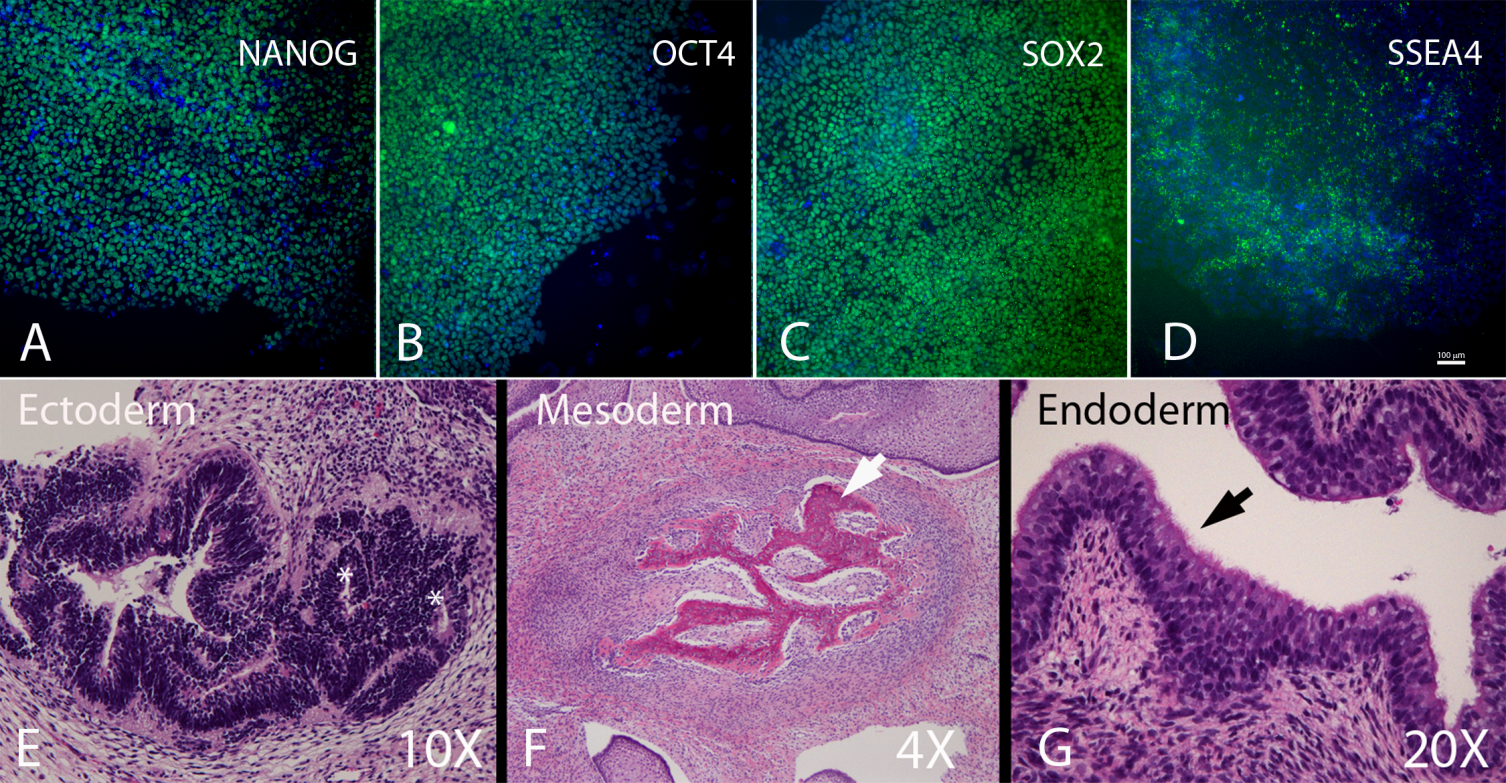
Pluripotency assayed by immunocytochemistry and teratoma formation. Baboon iPSCS were positive for the pluripotent transcription factors NANOG (A), OCT4 (B), SOX2 (C) and the cell surface marker SSEA4 (D). Following injection into NOD-SCID immunocompromised mice baboon iPSCs formed teratomas including tissues representative of all three germ layers. Neural rosettes of ectoderm (E, white asterisks), ossifying bone of mesoderm (F, white arrow) and ciliated lung epithelial of endoderm (G, black arrow).

An additional desirable characteristic of any protocol used for reprogramming adult somatic cells for potential therapeutic applications is that it be “footprint free”–i.e. that the method should not result in any permanent integration of exogenous genes or sequences [45]. To this end, we tested the Cytotune 2.0 kit (ThermoFisher Scientific) which employs Sendai virus-mediated delivery of genes encoding exogenous pluripotency factors. Sendai viruses are RNA viruses that replicate in the cytoplasm of the host cell without integrating into the genome, such that the virus is subsequently lost from the transduced cells after multiple passages. We followed the manufacturer’s instructions for the use of these virally delivered reprogramming factors to reprogram PBMCs from adult baboons and succeeded in deriving iPSCs with an efficiency of approximately 1:100,000 reprogrammed cells, which is similar to what has been reported for the derivation of iPSCs from human PBMCs [46]. We characterized these adult baboon PBMC-derived biPSC lines for expression of pluripotency factors, proper karyotype and competence to differentiate to form cell types characteristic of all three germ layers. These colonies expressed OCT4, NANOG, SOX2, and SSEA4 as assessed by immunocytochemistry (Fig 4A–4D) and possessed a normal 42XY male karyotype (S1 Fig). Additionally these cells formed tissues from all three germ layers when injected subcutaneously into immunocompromised mice including neural rosettes (Fig 4E, Ectoderm), ossifying bone (Fig 4F, Mesoderm) and ciliated endothelium (Fig 4G, Endoderm).

Sendai virus is an episomal RNA virus that is gradually lost in proliferating cells. To confirm that the virus has been elimated from the baboon iPSCS we isolated RNA from the cells and made cDNA. This was then probed using Taqman probes specific for the viral sequences. As a positive control we used baboon fibroblasts that had been reprogrammed just seven days previously as these are expected to still contain sendai virus. As shown in S5 Table despite loading higher levels of RNA in the baboon iPSC sample as evidenced by the HPRT1 threshhold cycle (Ct, 23.88 vs. 19.63) viral sequences were undetectable in the baboon iPSCs.

As mentioned above sendai virus is an RNA virus which does not have a DNA stage in its life cycle therefore any integration into the host genome would be exceedingly rare. However to discount this possibility we isolated genomic DNA from the baboon iPSCs and subjected it to qPCR for viral sequences as above. The HPRT1 probe we used does not recognize genomic DNA so instead we used a NANOG probe that is known to detect genomic DNA. As shown in S6 Table although genomic DNA is detected in the baboon iPSC sample no viral sequences were detected. Taken together these data indicate that the Sendai virus does not persist in these cells either episomally or integrated into the genome.

To determine if baboon iPSCs could differentiate into tissues from the three germ layers in vitro we used the trilineage differentiation kit (StemCell technologies). A single cell suspension was obtained using Accutase and plated onto matrigel, five (Endoderm, Mesoderm) or seven (Ectoderm) days later RNA was isolated, converted to cDNA and probed for genes respective of the different germ layers, pluripotent (OCT4 and SOX2), ectoderm (SOX2, NES, and PAX6), endoderm (SOX17 and FOXA2) and mesoderm (T also known as brachyury). As displayed in S2 Fig all three differentiation conditions resulted in a decrease in OCT4 expression, while both endoderm and mesoderm decreased the expression of SOX2. SOX2 is involved in neural differentiation so it is not surprising that this is higher in ectodermally differentiated cells. In addition to the increase in SOX2 expression NES and PAX6 were upregulated in ectoderm. Both SOX17 and FOXA2 were upregulated in endodermally differentiated conditions and T was upregulated 1000x in mesodermally differentiated cells. These results demonstrate that in addition to formation of all three germ layers in teratomas, baboon iPSCs can differentiate into tissues from all three germ layers in vitro as well.

## Discussion

Optimization of the efficacy and safety of methodologies associated with new cell-based therapies is needed to transition these methods to the clinic. Initial experiments in rodents, or in other small animal models such as pigs or rabbits will all yield informative results. However, differences in overall size of individual organs, or in general anatomy and physiology (particularly neuroanatomy and neurophysiology), or in genome sequence or epigenetic programming of different cell types between each of these species and humans may limit the extent to which such test data from these animal models will accurately predict clinical outcomes. Nonhuman primates (NHPs) provide the most clinically relevant animal models for preclinical biomedical research [47]. Among the NHP species available for research, baboons provide many unique attributes that render them the most informative preclinical model for testing cell-based therapeutic approaches [12].

Toward our goal of optimizing the baboon model, we previously generated ESC and iPSC lines from baboons [31, 33]. However, on the basis of our experience culturing pluripotent stem cells from rodents [34], other NHPs [35, 36] and humans [37–39], we have found the derivation and maintenance of baboon pluripotent stem cells to be the most challenging. To improve the utility and efficiency of baboon stem cells for preclinical studies, we first sought to identify standardized culture conditions that would maximize long-term maintenance of these cells in a pluripotent state while minimizing spontaneous differentiation. Previous reports showing that commercially prepared media often outperform media prepared with the same ingredients within individual laboratories [40] led us to test commercially prepared media from multiple sources to identify conditions that will support long-term culture of pluripotent baboon cells in a more consistent manner than did the baseline media prepared in our individual lab.

We tested four different commercially available media to determine which can best support the derivation and maintenance of pluripotent cells from baboons. We found that only one of these–Pluristem–can be effectively used to support the maintenance of baboon pluripotent cells in our hands. Pluristem is a proprietary medium for which information about the constituents is limited. Therefore a thorough comparison of the makeup of this media relative to that of the other commercial media tested, or to our baseline media to determine what is different about Pluristem is not possible.

Having determined that Pluristem best supports ongoing maintenance of biPSCs in a pluripotent state, we next determined if the use of Pluristem would improve the efficiency with which we can reprogram differentiated somatic cells from adult baboons to form new lines of biPSCs. We previously derived biPSCs from skin fibroblasts recovered from fetal baboons (Navara et al., 2013), but we have not succeeded in reprogramming skin fibroblasts from adult baboons–even when Pluristem media was used. Previous reports showed that iPSC lines can be generated from human adult PBMCs [46]. Therefore we tested the use of Pluristem media to support reprogramming of PBMCs from adult baboons to generate new lines of biPSCs. Adult PBMCs are less proliferative than adult skin fibroblasts during reprogramming, which may have contributed to our ability to successfully establish biPSC lines from the former but not from the latter. In addition, culture of biPSCs in Pluristem appeared to facilitate expression of SOX2 at higher levels than did culture of biPSCs in our baseline media, which may also have contributed to our ability to derive new lines of biPSCs from adult PBMCs in this media.

A final goal was to induce reprogramming of baboon somatic cells using a method that would result in no genomic footprint–i.e. a method that will not create any change in the sequence of the genomic DNA. We found that Sendai virus-mediated delivery of genes encoding exogenous pluripotency factors is an effective method by which to induce reprogramming without creating any genomic footprint. The biPSC lines derived from adult PBMCs all demonstrated appropriate pluripotent marker expression and normal baboon karyotypes, and generated tissues representative of all three germ layers when induced to form teratomas. Therefore, we have now developed reliable protocols for the derivation and maintenance of footprint-free pluripotent biPSC lines from a somatic cell type from adult baboons based on the use of Sendai virus-mediated induction of reprogramming and Pluristem culture media.

Importantly, PBMCs can be readily retrieved from live baboons via a simple, minimally invasive blood draw. This represents an ideal preclinical model that can be used to optimize the efficacy and safety of cell-based therapies involving patient-specific iPSCs. Thus, a blood sample from any individual baboon selected on the basis of an existing disease or debilitating phenotype or in which a specific disease or debilitation has been induced can be used to generate an iPSC line specific to that baboon. From the resulting biPSC line, the appropriate differentiated somatic cell type can be produced and, following purification, the differentiated cells can then be transplanted back into the same baboon from which the biPSC line was derived to facilitate testing of an individual-specific cell-based approach to reversing the relevant disease or debilitation. This model system will facilitate studies with primate cells to test and optimize protocols for the directed differentiation of specific cell types, purification and transplantation of those differentiated cells to appropriate disease-specific locations in the body, tracking of the transplanted cells to assess their persistence and proliferation *in situ*, recovery of the transplanted cells at various post-transplantation time points to assess patterns of gene expression and epigenetic programming, or continued observation of the transplanted cells *in situ* to assess the extent to which they mitigate the specific disease or debilitation being treated, and to ensure the absence of undesirable off-target effects such as tumorigenesis. Thus, the use of this primate model that so closely resembles the human will yield the most clinically relevant information regarding the efficacy and safety of each specific cell-based therapeutic protocol that can then be advanced to use in the clinic with maximum confidence.

## Conclusions

In this study we identified a commercially available culture medium that enhanced the consistent maintenance of baboon iPSCs in a pluripotent state and facilitated, for the first time, derivation of iPSCs from adult baboon somatic cells. These results combined with our previous reports characterizing neural differentiation from baboon iPSCs (Grow et al., 2016a,b) clear the way for *in vivo* testing of stem cell derived therapies in baboons–the most clinically relevant model system for optimizing the efficacy and safety of cell-based therapies.

## Supporting information

S1 Fig Karyotype of PBMC derived iPSC line. G-band analysis revealed a normal male baboon 42XY karyotype.
(TIF)

S2 Fig Trilineage differentitation of baboon iPSCs. Baboon iPSCs differentiated five (Endoderm, Mesoderm) or seven (Ectoderm) days in vitro expressed genes representative of the three germ layers, ectoderm (SOX2, NES, and PAX6), endoderm (SOX17 and FOXA2) and mesoderm (T also known as brachyury).
(PDF)

S1 Table Antibodies and dilutions. (PDF)

S2 Table PCR primers. (PDF)

S3 Table Media constituents. (PDF)

S4 Table Data for Fig 3. (PDF)

S5 Table Absence of persistent Sendai virus in baboon iPSCs. (PDF)

S6 Table No integration of Sendai virus in the genome of baboon iPSCs. (PDF)

S7 Table Ct values for technical reps for S5 Table. (PDF)

S8 Table Ct values for technical reps for S6 Table. (PDF)

S9 Table Ct values for S2 Fig. (PDF)

S1 File Arrive guidelines. (PDF)
