# Supplementary figures and images for: Optimization of culture conditions for the derivation and propagation of baboon (Papio anubis) induced pluripotent stem cells
Source: PLoS One. 2018 Mar 1;13(3):e0193195. doi: 10.1371/journal.pone.0193195 (PMC5832232; doi:10.1371/journal.pone.0193195)

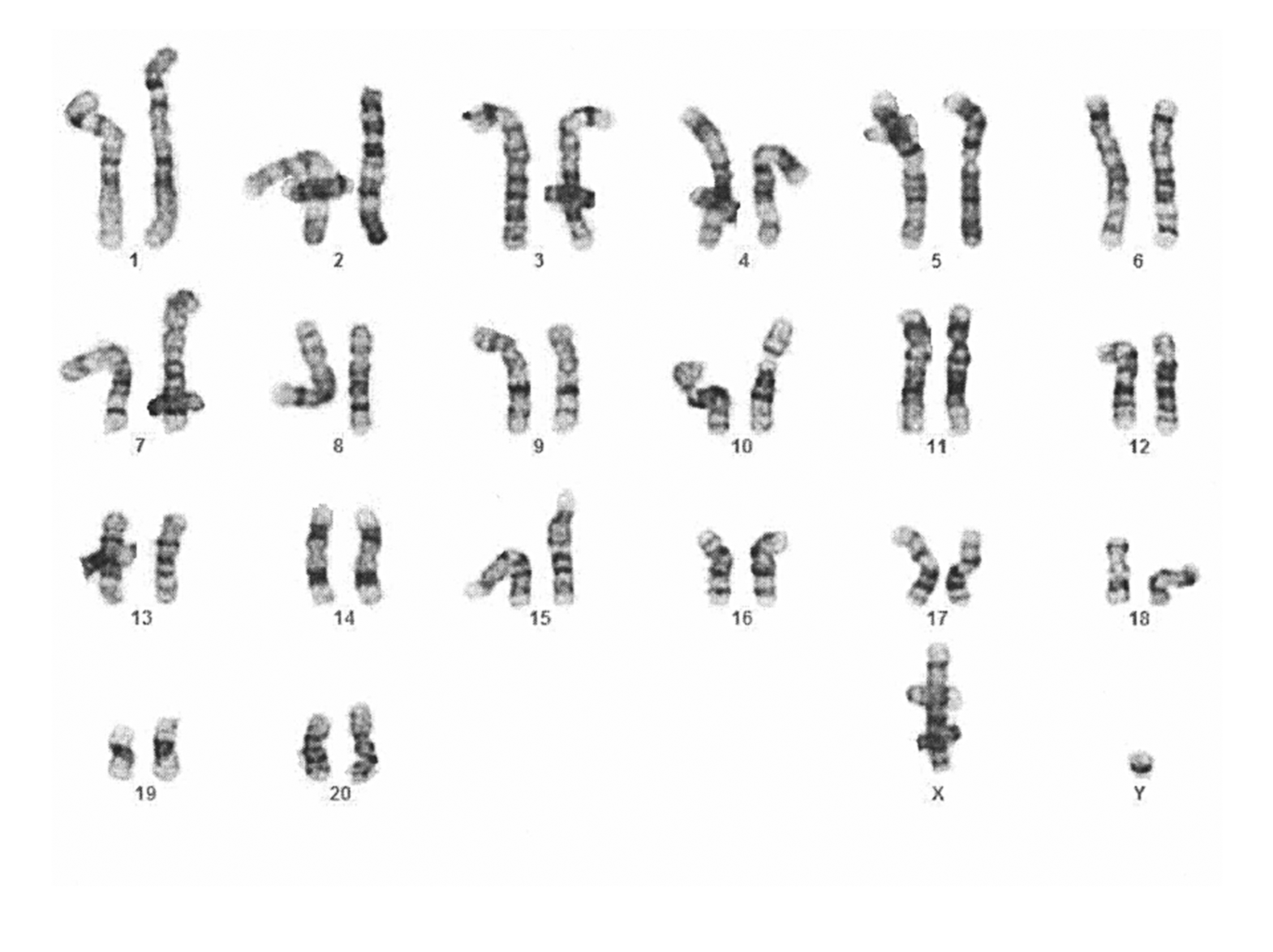

Supplement: S1 Fig — G-band analysis revealed a normal male baboon 42XY karyotype. (TIF) [file pone.0193195.s001.tif]

Trilineage differentiation of Baboon iPSCs

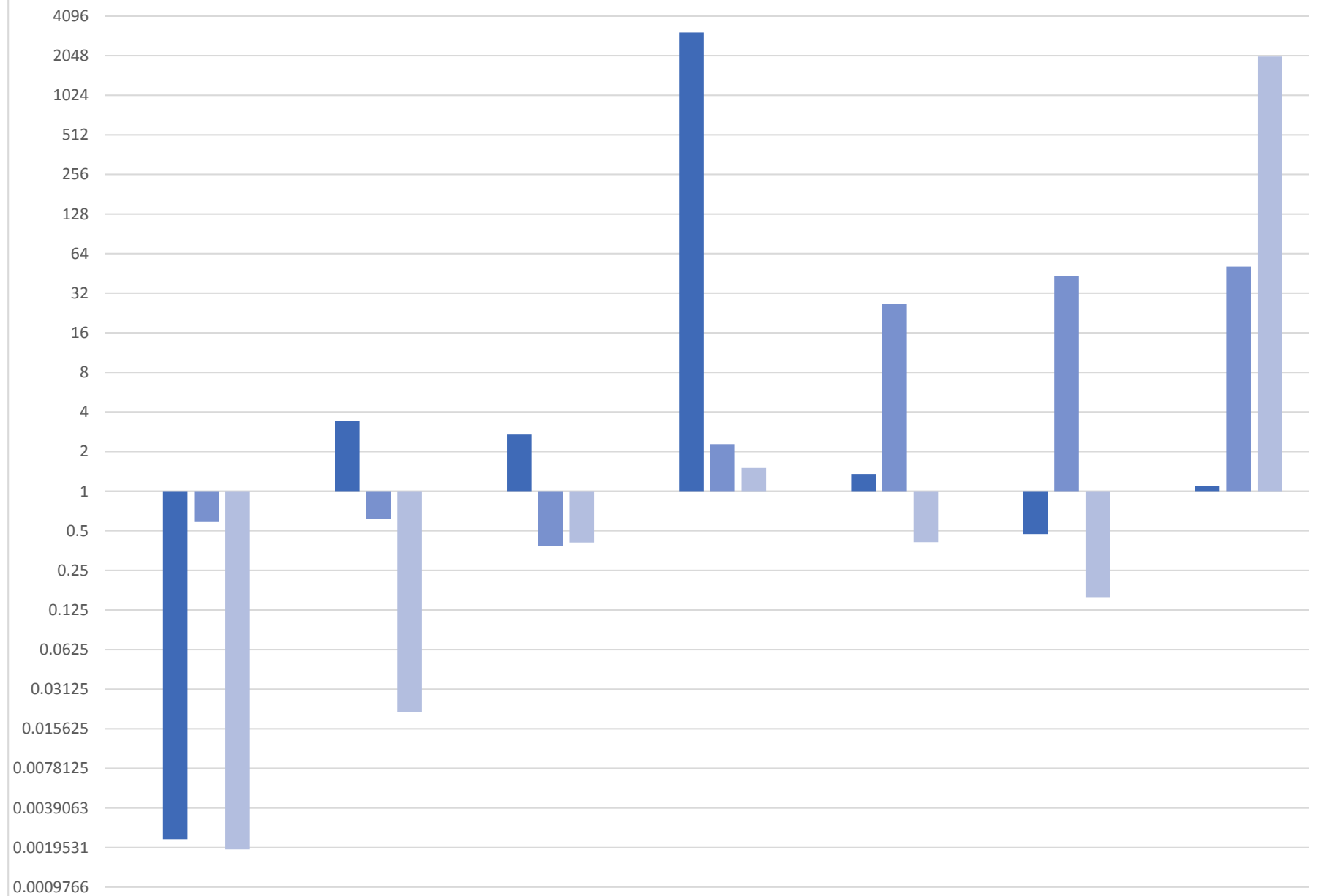

Supplement: S2 Fig — Baboon iPSCs differentiated five (Endoderm, Mesoderm) or seven (Ectoderm) days in vitro expressed genes representative of the three germ layers, ectoderm (SOX2, NES, and PAX6), endoderm (SOX17 and FOXA2) and mesoderm (T also known as brachyury). (PDF) [file pone.0193195.s002.pdf]
