# Supplementary material for: Optimization of culture conditions for the derivation and propagation of baboon (Papio anubis) induced pluripotent stem cells
Source: PLoS One. 2018 Mar 1;13(3):e0193195. doi: 10.1371/journal.pone.0193195 (PMC5832232; doi:10.1371/journal.pone.0193195)
Supplement: S1 Table — (PDF) [file pone.0193195.s003.pdf]

S1 Table. Antibodies and dilutions.

Target	Company	Product Number	Dilution
OCT4	Sant Cruz Biotechnology	sc-5279	1:100
NANOG	Abcam	ab2163	1:150
SOX2	Cell Signaling Technologies	3579	1:400
SSEA4	Sant Cruz Biotechnology	sc-21704	1:5
TRA181	Sant Cruz Biotechnology	sc-21706	1:5
