# Supplementary material for: Optimization of culture conditions for the derivation and propagation of baboon (Papio anubis) induced pluripotent stem cells
Source: PLoS One. 2018 Mar 1;13(3):e0193195. doi: 10.1371/journal.pone.0193195 (PMC5832232; doi:10.1371/journal.pone.0193195)
Supplement: S2 Table — (PDF) [file pone.0193195.s004.pdf]

S2 Table. PCR primers.

Gene	Forward	Reverse
B2M	CTTTCTGGCCTGGAGGCTATC	GGCTTTCCATTCTCTGGTGGA
OCT4	AGGAGAAGCTGGAGCAAAACC	TGGCTGAACACCTTCCCAAATA
NANOG	TGAGATGCCTCACACGGAG	GCTGGGCAGAAGAGAACACA
SOX2	GTTACGCGCATATGAACGGC	CGAGCTGGTCATGGAGTTGT
NES	AGCGTTGGAACAGAGGTTGG	CACAGCCAGCTGGAACTTTTC
PAX6	CTAATGGGCCAGTGAGGAGC	TTCTCAGATTCCTATGCTGATTGG
SOX17	GTGGACCGCACGGAATTTC	GGAGATTCACACTGGAGTCA
FOXA2	CGGTCACGAACAAAACGGG	AGAGGAGTTCATAATGGGCCG
T	CCTTGATGCAAAGGAAAGAAGCG	TCCAGGAAGAAACCACCCCC
