# Supplementary material for: Optimization of culture conditions for the derivation and propagation of baboon (Papio anubis) induced pluripotent stem cells
Source: PLoS One. 2018 Mar 1;13(3):e0193195. doi: 10.1371/journal.pone.0193195 (PMC5832232; doi:10.1371/journal.pone.0193195)
Supplement: S3 Table — (PDF) [file pone.0193195.s005.pdf]

S3 Table. Media constituents.

Media	mTeSR-1	TeSR-E8	Pluristem*	ReproFF
	DMEM/F12	DMEM/F12	DMEM/F12	?
	NaHCO3	NaHCO3 (543 mg/L)		?
	l-Ascorbic Acid	l-Ascorbic Acid-2-phosphate magnesium (64mg/L)		?
	Selenium	Sodium Selenium (14 ug/L)	Selenium	?
	Transferrin	Transferrin (10.7 mg/L)	Transferrin	?
	Insulin	Insulin (19.4 ug/ml)	Insulin	?
	FGF-2	FGF-2 (100 ug/L)	FGF-2 (10 ug/L)	?
	TGF-B	TGF-B (2 ug/L)	TGF-B (0.5 ug/L)	?
	BSA			?
	Glutathione			?
	Trace elements			?
	BME			?
	Pipecolic Acid			?
	GABA			?
	Lithium Chloride			?
	Defined Lipids		Defined Lipids (1x)	?
			Dorsomorphin (50 nM)	?
			Activin A (2.5-5 ug/L)	?
			HSA (0.1%)	?
			Glutamine (2mM)	?

known component and concentration

known component

*Likely components
