# Supplementary material for: Optimization of culture conditions for the derivation and propagation of baboon (Papio anubis) induced pluripotent stem cells
Source: PLoS One. 2018 Mar 1;13(3):e0193195. doi: 10.1371/journal.pone.0193195 (PMC5832232; doi:10.1371/journal.pone.0193195)
Supplement: S4 Table — (PDF) [file pone.0193195.s006.pdf]

S4 Table. Ct values for Fig 3.

Conditioned Media Baseline

	B2M	OCT4	NANOG	SOX2
Expt 1	17.86	17.12	18.15	20.72
Expt 2	17.98	17.45	18.64	21.32
Expt 3	17.49	16.95	18.43	20.51
Expt 4	18.07	18.02	18.5	21.67

Pluristem

	B2M	OCT4	NANOG	SOX2
Expt 1	18.52	18.05	18.89	21.1
Expt 2	18.62	17.68	19.23	20.81
Expt 3	18.82	17.47	19.27	21.39
Expt 4	18.05	17.92	18.22	20.54
