# Supplementary material for: Optimization of culture conditions for the derivation and propagation of baboon (Papio anubis) induced pluripotent stem cells
Source: PLoS One. 2018 Mar 1;13(3):e0193195. doi: 10.1371/journal.pone.0193195 (PMC5832232; doi:10.1371/journal.pone.0193195)
Supplement: S5 Table — (PDF) [file pone.0193195.s007.pdf]

S5 Table. Absence of persistent Sendai virus in baboon iPSCs

Sample	Average Ct
Positive Control	
HPRT1	23.88
SEV	15.93
KOS	26.42
CMYK	22.94
KLF4	22.03
Baboon iPSC	
HPRT1	19.63
SEV	N.D.
KOS	N.D.
CMYK	N.D.
KLF4	N.D.
No Template	
HPRT1	N.D.
SEV	N.D.
KOS	N.D.
CMYK	N.D.
KLF4	N.D.

N.D. = not detected
