# Supplementary material for: Optimization of culture conditions for the derivation and propagation of baboon (Papio anubis) induced pluripotent stem cells
Source: PLoS One. 2018 Mar 1;13(3):e0193195. doi: 10.1371/journal.pone.0193195 (PMC5832232; doi:10.1371/journal.pone.0193195)
Supplement: S6 Table — (PDF) [file pone.0193195.s008.pdf]

S6 Table. No integration of Sendai virus in the genome of baboon iPSCs

Sample	Average Ct
Positive Control	
NANOG	29.88
SEV	16.01
KOS	27.09
CMYK	22.51
KLF4	23.50
Baboon iPSCs	
NANOG	26.24
SEV	N.D.
KOS	N.D.
CMYK	N.D.
KLF4	N.D.

N.D. = not detected
