# Supplementary material for: Optimization of culture conditions for the derivation and propagation of baboon (Papio anubis) induced pluripotent stem cells
Source: PLoS One. 2018 Mar 1;13(3):e0193195. doi: 10.1371/journal.pone.0193195 (PMC5832232; doi:10.1371/journal.pone.0193195)
Supplement: S7 Table — (PDF) [file pone.0193195.s009.pdf]

Ct values for technical reps S5 Table.

Positive Control	HPRT1	SEV	KOS	CMYK	KLF4
Technical Rep 1	23.88587	15.97409	25.90277	22.87782	21.95252
Technical Rep 2	24.06782	15.88355	27.0027	22.99329	22.01403
Technical Rep 3	23.69288	27.26427	26.35552	35.70842	22.1291

Baboon iPSC	HPRT1	SEV	KOS	CMYK	KLF4
Technical Rep 1	20.08551	N.D.	N.D.	N.D.	N.D.
Technical Rep 2	19.69429	N.D.	N.D.	N.D.	N.D.
Technical Rep 3	19.12404	N.D.	N.D.	N.D.	N.D.

No Template	HPRT1	SEV	KOS	CMYK	KLF4
Technical Rep 1	N.D.	N.D.	N.D.	N.D.	N.D.
Technical Rep 2	N.D.	N.D.	N.D.	N.D.	N.D.
Technical Rep 3	N.D.	N.D.	N.D.	N.D.	N.D.

N.D. = not detected. A technical rep more than 3 cycles different from the average of the other two technical reps was considered an error and ignored.
