# Supplementary material for: Optimization of culture conditions for the derivation and propagation of baboon (Papio anubis) induced pluripotent stem cells
Source: PLoS One. 2018 Mar 1;13(3):e0193195. doi: 10.1371/journal.pone.0193195 (PMC5832232; doi:10.1371/journal.pone.0193195)
Supplement: S8 Table — (PDF) [file pone.0193195.s010.pdf]

Ct values for technical reps S6 Table.

Positive Control	NANOG	SEV	KOS	CMYK	KLF4
Technical Rep 1	29.39142	16.27601	26.88888	23.5176	23.01669
Technical Rep 2	29.57634	15.82531	26.82784	23.55842	22.62257
Technical Rep 3	30.66354	15.91514	27.56494	23.43613	21.91787

Baboon iPSC Genomic DNA	NANOG	SEV	KOS	CMYK	KLF4
Technical Rep 1	26.94623	N.D.	N.D.	N.D.	N.D.
Technical Rep 2	25.77532	N.D.	N.D.	N.D.	N.D.
Technical Rep 3	26.02016	N.D.	N.D.	N.D.	N.D.
