# Supplementary material for: Optimization of culture conditions for the derivation and propagation of baboon (Papio anubis) induced pluripotent stem cells
Source: PLoS One. 2018 Mar 1;13(3):e0193195. doi: 10.1371/journal.pone.0193195 (PMC5832232; doi:10.1371/journal.pone.0193195)
Supplement: S9 Table — (PDF) [file pone.0193195.s011.pdf]

Ct values for S2 Figure

Pluripotent	HPRT	OCT4	SOX2	NESTIN	PAX6	SOX17	FOXA2	T
Technical Rep 1	18.91478	12.73304	18.2409	19.62981	28.82896	23.8229	22.9652	30.54389
Technical Rep 2	18.2678	12.78222	17.45946	19.36095	28.85317	23.53583	22.42593	31.51155
Technical Rep 3	18.06661	12.60524	17.83647	21.81421	28.4202	23.80138	22.71646	30.12008

Ectoderm	HPRT	OCT4	SOX2	NESTIN	PAX6	SOX17	FOXA2	T
Technical Rep 1	19.86724	23.00622	16.99774	18.78698	18.01881	24.09042	24.42718	29.5016
Technical Rep 2	19.04234	22.21484	16.70148	18.91803	18.12485	24.14227	24.71437	30.81405
Technical Rep 3	18.93877	21.86814	17.12406	18.7114	17.80637	24.23329	24.81458	34.08133

Mesoderm	HPRT	OCT4	SOX2	NESTIN	PAX6	SOX17	FOXA2	T
Technical Rep 1	18.84861	39.22387	22.96089	21.19192	29.67801	24.60119	25.37571	22.84632
Technical Rep 2	17.79291	21.48611	22.99745	19.90143	27.15497	24.7211	24.83654	17.92066
Technical Rep 3	17.90904	21.55362	23.63439	20.1985	26.81117	25.00024	25.23054	17.77508
	18.18352	21.51987	23.19757	20.43062	26.98307	24.77418	25.1476	17.84787

Endoderm	HPRT	OCT4	SOX2	NESTIN	PAX6	SOX17	FOXA2	T
Technical Rep 1	19.11546	14.2584	19.63887	21.81421	29.52824	19.69242	18.29554	30.12008
Technical Rep 2	19.64856	14.56878	19.47438	21.73194	28.00147	20.18413	18.47433	25.97022
Technical Rep 3	19.22268	14.31677	19.29729	21.457	27.75807	19.82191	18.06479	25.96567

A technical rep more than 3 cycles different from the average of the other two technical reps was considered an error and ignored.
